# Bmp7 Regulates the Survival, Proliferation, and Neurogenic Properties of Neural Progenitor Cells during Corticogenesis in the Mouse

**DOI:** 10.1371/journal.pone.0034088

**Published:** 2012-03-26

**Authors:** Aikaterini Segklia, Eve Seuntjens, Maximilianos Elkouris, Sotiris Tsalavos, Elke Stappers, Thimios A. Mitsiadis, Danny Huylebroeck, Eumorphia Remboutsika, Daniel Graf

**Affiliations:** 1 Institute of Immunology, Biomedical Sciences Research Center Alexander Fleming, Vari, Hellas-Greece; 2 Laboratory of Molecular Biology (Celgen), Center for Human Genetics, K.U.Leuven, Leuven, Belgium; 3 Department of Molecular and Developmental Genetics, VIB, K.U.Leuven, Leuven, Belgium; 4 Institute of Molecular Biology and Genetics, Biomedical Sciences Research Center Alexander Fleming, Vari, Hellas-Greece; 5 Faculty of Medicine, Institute of Oral Biology, University of Zurich, Zurich, Switzerland; University of Nebraska Medical Center, United States of America

## Abstract

Bone morphogenetic proteins (BMPs) are considered important regulators of neural development. However, results mainly from a wide set of *in vitro* gain-of-function experiments are conflicting since these show that BMPs can act either as inhibitors or promoters of neurogenesis. Here, we report a specific and non-redundant role for BMP7 in cortical neurogenesis *in vivo* using knockout mice. Bmp7 is produced in regions adjacent to the developing cortex; the hem, meninges, and choroid plexus, and can be detected in the cerebrospinal fluid. *Bmp7* deletion results in reduced cortical thickening, impaired neurogenesis, and loss of radial glia attachment to the meninges. Subsequent *in vitro* analyses of E14.5 cortical cells revealed that lack of Bmp7 affects neural progenitor cells, evidenced by their reduced proliferation, survival and self-renewal capacity. Addition of BMP7 was able to rescue these proliferation and survival defects. In addition, at the developmental stage E14.5 Bmp7 was also required to maintain *Ngn2* expression in the subventricular zone. These data demonstrate a novel role for Bmp7 in the embryonic mouse cortex: Bmp7 nurtures radial glia cells and regulates fundamental properties of neural progenitor cells that subsequently affect Ngn2-dependent neurogenesis.

## Introduction

Embryonic brain development is based on the sequential generation and differentiation of neuroepithelial precursor cells. A strict temporal sequence controls the development of the various cell types in the mouse brain: initially the neurons are formed, followed by the astrocytes and oligodendrocytes [1]. In the telencephalon, distinct areas may act as signalling centers that control these developmental steps. It has been well-established that Bone Morphogenetic Proteins (BMP) control neural development [2].

Members of the large BMP subgroup of the Transforming Growth Factor-β (TGF-β) family of secreted signalling proteins have important pleiotropic functions not only during embryogenesis but also after birth [3], [4]. BMPs signal through a receptor complex consisting of two type I serine-threonine kinase receptors (e.g. Activin receptor-like kinase (Alk)1, Alk2, Alk3 (also known as BmprIa) or Alk6 (BmprIb)) and two type II receptors (BmprII or ActRII) [5]. The type I receptors in the ligand-activated receptor complex phosphorylate the intracellular BMP-Smad effector proteins (Smad1, 5 and 8) [6] but also activate non-BMP-specific signal transduction pathways such as MAPK/PI3K/Akt [5]. BMP signalling activity *in vivo* is highly regulated at several levels of the pathway, including extracellularly where secreted BMP-binding proteins like Noggin, Chordin, and Gremlin act as BMP antagonists [7]. Binding affinities to antagonists and receptors differ between the various members of the BMP subgroup ligands [8] and contribute to the precise spatio-temporal regulation of BMP biological activity *in vivo* [8]. However, despite intensive research in the BMP field over the last 20 years the cell-type specific requirements for individual members of the BMP subgroup in complex tissue interactions during embryogenesis and organogenesis are still largely unknown.

Genes encoding BMPs, BMP antagonists and BMP receptors are expressed in the developing and adult brain and their encoded proteins are implicated in various aspects of neural development. For example, BMPs are involved in the organization of the medial-lateral axis of the developing telencephalon and its division into clearly defined regions [2], and gain-of-function experiments have shown that they control glial cell fate [9]. BMPs can promote astrocyte formation at the expense of neurons in cell culture, suggesting their important regulatory role in this aspect of correct brain development [10]. In support of this, over-expression of *Bmp4* in neurons of late-gestation mouse embryos increases the number of astrocytes at the expense of oligodendrocytes [11]. However, BMPs do not always promote the glial cell fate at the expense of neurons. For example, BMP-mediated signalling via Smad4 is required to initiate neurogenesis from adult neural stem cells and suppress the alternative fate of oligodendrogliogenesis [12]. BMPs also promote sensory neurogenesis at the expense of gliogenesis in trunk neural crest cells [13] and act in synergy with Wnt to maintain neural crest stem cells [14]. BMPs also appear to regulate neuronal migration: overexpression of *Bmp7* in the developing cerebral cortex does not only induce premature radial glia differentiation but indeed also impairs neuronal migration [15]. BMPs have been implicated as pro-survival factor for neurons. For example, BMP7 reduces the effects of ischemia-induced brain infarction [16], promotes cell survival in cerebellar granule cells [17] and has a neuroprotective function on cultured primary cortical cells [18]. *In vivo* studies are now required to elucidate the functions of Bmp7 during mouse brain development.

Loss of *Bmp7* in the mouse causes defects in lens induction, skeleton, kidney, palate and teeth [19], [20], [21] and is perinatal lethal, which has been attributed to uremia due to the non-functional kidneys [19], [20]. Double mutants for *Bmp5;Bmp7* and *Bmp6;Bmp7* show more severe phenotypes and die by mid-gestation [22] suggesting that some functional redundancy and/or compensation might exist amongst these BMPs. Here we describe a novel and non-redundant role for BMP7 in the developing cortex through new studies in *Bmp7* knockout mouse embryos. We find that Bmp7 is required for the proper architecture of the developing mouse brain cortex and acts as a trophic and survival factor for cortical progenitor cells.

## Materials and Methods

### Mice

The BMP7^wt/Δ^ allele used in this study was derived by deleting a BMP7^wt/flx^ allele in the germline [21]. The *Bmp7*-lacZ reporter allele has been described elsewhere [23]. Both mouse lines were backcrossed for more than 8 generations into the C57Bl6/J background. Mice were maintained under specific pathogen-free conditions at the animal facility of the Biomedical Sciences Research Center Alexander Fleming. All animals were handled in strict accordance with good animal practice as defined by the Animals Act 160/03.05.1991 applicable in Greece, revised according to the 86/609/EEC/24.11.1986 EU directive regarding the proper care and use of laboratory animals and in accordance with the Greek License for Animal Experimentation at the BSRC Alexander Fleming (Prot. No. 767/28.02.07) issued after protocol approval by the Animal Care and Use Committee of the BSRC Alexander Fleming (Prot. No. 1243/26.03.2007).

### Labeling with BrdU

Incorporation of BrdU was done by i.p. injection of 10 µl per g body weight of the pregnant mother using a 10 mM BrdU solution (Sigma). Mice were injected 2 hrs before they were sacrificed.

### RT-PCR

Total RNA was extracted using Tri Reagent (MRC, Cinncinati, USA) according to the manufacturers' instructions. For cDNA synthesis 2 µg of total RNA were primed with oligo-dT and reverse transcribed using a MuMLV Reverse Transcriptase (New England Biolabs). For semi-quantitative RT-PCR 1∶4 serial dilutions of cDNA were subjected to amplification using Taq Polymerase (Invitrogen). Primers used: *Ngn2*: fwd (5′-GGACTGACTGACAGACAACCACG-3′), rev (5′-CCTCCTCTTCCTCCTTCAACTCC-3′); *Pax6*: fwd (GCTTCATCCGAGTCTTCTCCGTTAG), rev (CCATCTTGCTTGGGAAATCCG); *Gapdh*: fwd (5′-TCTTCTTGTGCAGTGCC-3′), rev (5′-ACTCCACGACATACTCAGC-3′).

### LacZ staining

OCT (BDH)-embedded cortex sections were fixed in 2% formaldehyde, 0.2% gluteraldehyde, 0.01% sodium deoxycholate, 0.02% Nonidet-P40 (NP40) in PBS for 5 min, washed with 2 mM MgCl_2_, and stained overnight in X-gal solution [0.1 M phosphate pH 7.3, 2 mM MgCl_2_, 0.01% sodium deoxycholate, 0.02% NP40, 5 mM K_3_Fe(CN)_6_, 5 mM K_4_Fe(CN)_6_] supplemented with 1 mg X-Gal (Promega)/ml X-gal staining solution at 37°C in the dark overnight. The following day, sections were washed, counter-stained with Nuclear Fast Red (Sigma) and mounted with water-based Mowiol 4–88 (Sigma). Sections were documented using a Nikon Eclipse microscope.

### Histology, Immunohistochemistry and Immunofluorescence

OCT (BDH)-embedded tissues were snap-frozen in liquid nitrogen steam. 6 µm cryostat sections were placed on gelatin-coated slides, air-dried, fixed in 10% formalin for 10 min and washed in phosphate-buffered saline (PBS) at RT. For paraffin-embedding tissues were first fixed in 4% paraformaldehyde in PBS at 4°C overnight, then embedded in paraffin and sectioned at 5–6 µm on TESPA or Superfrost Plus slides (BDH).

For histological analysis, paraffin sections were stained with hematoxylin and eosin (H&E) following standard procedures. Briefly, paraffin processed cortex sections were re-hydrated, the cell nuclei were stained with hematoxylin, passed through acid alcohol for stain differentation and quickly rinsed in Scott's solution (blueing agent). The cell membranes were stained with Eosin for 2 min. Sections were mounted with xylene-based mounting medium.

For immunohistochemistry, the cryostat or paraffin sections were incubated overnight with the following antibodies: mouse anti-Map2 (1∶200, Sigma), rabbit anti-Tbr2 (1∶500, Millipore), rabbit anti-pMAPK (1∶200, Cell Signaling Technologies), rabbit anti-pSmad1/5/8 (1∶100, Cell Signaling Technologies). Secondary antibodies used were biotinylated anti-mouse IgG (1∶1000, Jackson Immunosearch) or biotinylated anti-rabbit IgG (1∶1000, Jackson Immunosearch) and detection was done with Streptavidin-conjugated horse radish peroxidase (HRP).

For immunofluorescence, cryotstat or paraffin sections were incubated with the following primary antibodies: mouse anti-BrdU (1∶100, DSHB, Univ. Iowa), rabbit anti-PH3 (1∶150, Santa Cruz), mouse anti-PCNA (1∶400, Santa Cruz), rabbit anti-Tbr1 (1∶3000, Millipore), rat anti-Ctip2 (1∶300, Abcam), mouse anti-RC2 (1∶100, DSHB, Univ. Iowa), rabbit anti-BMP7 (1∶100, Peprotech). Secondary antibodies used were goat anti-mouse IgG Alexa555 (1∶1000, Invitrogen), goat anti-mouse Alexa488 IgG2A (1∶1000, Invitrogen), donkey anti-rat CY2 (1∶600, Jackson Immunosearch), donkey anti-goat CY3 (1∶600, Jackson Immunosearch), donkey anti-rabbit CY3 (1∶600, Jackson Immunosearch), donkey anti-rabbit Alexa488 (1∶1000, Invitrogen). For all stainings, a minimum of three control and three mutants were analyzed for each marker at each stage.

### *In Situ* hybridization on sectioned tissues

Paraffin sections were prepared as described above and the *in situ* procedure was carried out using an automated platform (Discovery Xt, Ventana Medical Systems, Roche). Details of the methods are available upon request. A minimum of three control and three mutant embryos were analyzed for each probe at each stage. Plasmids for the following *in situ* probes (150 ng used for each reaction) were obtained: *Ngn1* and *Ngn2* from Q. Ma (Caltech, US), *NSCL2* from F. Guillemot (NIMR London, UK), *NeuroD* from J. Lee (U. Colorado, US), *Svet1* from V. Tarabykin (Charité Univ., Berlin, Germany), *Reelin* from A. Goffinet (UCL, Belgium), *Pax6* from K. Eto (Tokyo Univ., Japan) and *GAD67* from B. Condie (Univ. Georgia, US). Following the procedure slides were dehydrated and mounted with Eukitt (Sigma).

### Western Blot

Ten µg of protein extract was loaded on acrylamide gel for SDS-PAGE electrophoresis. Western blotting was carried out on nitrocellulose membrane (Whatman) blocked with PBS/0.05% Tween-20/2.5% BSA and probed with rabbit anti-BMP2 (Peprotech), mouse anti-BMP4 (Peprotech), goat anti-BMP5 (Santa Cruz), goat anti-BMP6 (Santa Cruz), rabbit anti-BMP7 (Peprotech) overnight in PBS/0.05% Tween-20, 1% BSA. Membranes were washed three times for 10 min each with PBS/0.05% Tween-20 and incubated with the appropriate secondary IgGs coupled to HRP. Membranes were visualized using the ECL Plus detection kit (Amersham) on Biomax film (Kodak). To assess equal protein loading, membranes were stripped and re-probed with anti-GAPDH (Sigma).

### Detection of Bmp7 protein in cerebrospinal fluid

Embryonic cerebrospinal fluid (CSF) was aspirated from several embryos at E12.5, E13.5 or E14.5 and pooled. Protein content was measured using Nanodrop. Ten µg of protein (corresponding to about 5 µl of CSF) was boiled in 5×Loading Dye (62.5 mM Tris-HCl pH 6.8, 2% SDS, 25% Glycerol, 0.01% Bromophenol Blue) and subjected to electrophoresis in 12% SDS-PAGE and blotted onto a nitrocellulose filter. The blot was blocked in TBS/0.05%Tween-20/2.5% non-fat milk powder, and primary antibody (anti-BMP7, 1∶500) was applied overnight at 4°C. After washing in TBS/0.05% Tween-20 and incubation with secondary antibody (ant-rabbit-HRP, 1∶25000), the blot was again washed and then visualized using ECL Plus detection kit (Amersham) on Biomax film (Kodak).

### Cultures of Neuroepithelial cells and Neurosphere assays

For the isolation of neuroepithelial cells, the uterus of the pregnant mice was dissected at E14.5, the embryos were removed and the embryo heads were excised. With the use of fine forceps, the epidermis, the mesenchyme and the meninges were removed in order to reveal the telencephalon. The neuroepithelium of the dorsal telencephalon was triturated using a plugged sterile flame-prepared drawn-out Pasteur pipette in neural stem cell medium, consisting of DMEM/F-12 (1∶1) (GIBCO), supplemented with 2 mM glutamine (GIBCO), 1% Penicillin/Streptomycin (GIBCO), 0,6% glucose (Sigma), 25 mg insulin/ml (Sigma), 9.6 mg putrescine/ml (Sigma), 6.3 ng progesterone/ml (Sigma), 5.2 ng sodium selenite/ml (Sigma), 100 mg transferrin/ml (Sigma), 20 ng basic fibroblast growth factor/ml (bFGF; GIBCO), and 20 ng epidermal growth factor/ml (EGF; GIBCO).

For *in vitro* stimulation, cells were transferred to DMEM/F-12 (1∶1) medium (GIBCO), supplemented with 2 mM glutamine, 1% Penicillin/Streptomycin, and further cultured on polylysine-coated slides at a seeding density of 5×10^4^ cells/ml. For stimulation, recombinant human BMP7 (Peprotech) was added at 50 ng/ml for 24 hrs in normal culture conditions.

For the neurosphere assay, a single cell suspension was seeded at a clonal density of 1×10^4^ cells/ml on 100 mm tissue culture ultra-low attachment plates (Corning). After 8 days in culture the number of primary spheres and the number of cells per sphere were examined. For analysis, primary spheres were collected after 7 days in culture, fixed in 4% paraformaldehyde in PBS for 7 min on ice, and then washed three times in PBS on ice.

## Results

### BMP7 is necessary for the correct regulation of cortical plate size

**Figure 1:**
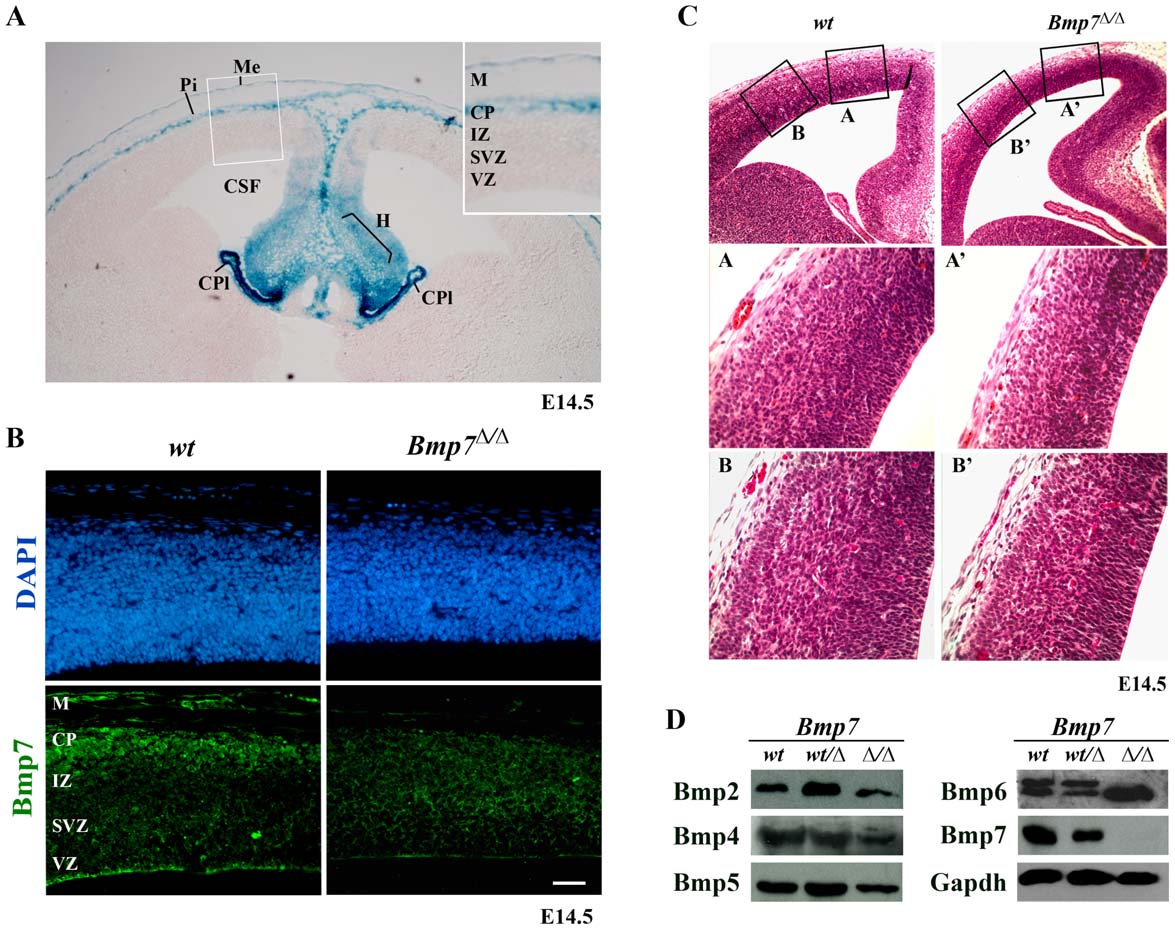
*Bmp7* null embryos suffer from microencephaly. (A) *Bmp7* expression as monitored by lacZ-reporting in the mouse E14.5 cortex. Strong expression is seen in the medial regions, such as hem (H) and choroid plexus (CPl), the pial membrane (Pi) and meninges (Me). No expression is apparent in the ventricular (VZ), the subventricular (SVZ), and intermediate (IZ) zones, and the cortical plate (CP) (inlay). (B) Bmp7 protein (lower panels; the staining with DAPI is shown in the upper panels) is observed in the marginal zone (M), the CP and VZ of wild-type embryos (E14.5) and is lost in the *Bmp7*-deficient mouse embryo. (C) *Bmp7*-deletion results in microencephaly with a thinner cortex and a less clearly defined cortical plate, most prominent in medial (A, A′) and lesser in more lateral regions. (D) Other Bmp proteins present in the developing neocortex are not significantly altered following *Bmp7* deletion. The anti-Bmp6 antibody used here appears to cross-react with Bmp7, resulting in a second upper band, which is lost upon *Bmp7* ^Δ/Δ^ deletion.

Corticogenesis is guided by a combination of transcription factors acting downstream of extrinsic cues that establish within the cortex the identity, delineation and size of its domains. While BMPs have been suggested to be important in several major patterning pathways during embryogenesis their role in cortical development has not been well defined. To understand the role of BMP7 in the embryonic cortex, we first investigated Bmp7 gene expression in wild-type (wt) embryos, using a *Bmp7*-lacZ reporter mouse [23]. In E14.5 embryos we observed strong lacZ staining in medial regions of the brain, such as the hem and the choroid plexus, and in the pial membrane and the meninges (Fig. 1A). No *Bmp7* expression was evident in the ventricular, the subventricular and the intermediate zones of the cortex at this developmental stage. As the meninges and choroids plexus are essential for cell feeding and as substrates for cell migration [24], [25] we hypothesized that Bmp7 could regulate these cellular aspects of neocortical development. We therefore assayed for the presence of Bmp7 protein, using staining with an anti-Bmp7 antibody, in the proliferating zones and found that apart from the meninges where *Bmp7* mRNA is clearly expressed, Bmp7 protein was present also in the marginal zone and the ventricular zone at E14.5 (Fig. 1B). This observation indicates that the Bmp7 produced in the choroid plexus (similar to Gdf7/Bmp12, see [26]) could reach the ventricular zone through the cerebrospinal fluid (CSF). To test this idea, we assayed for Bmp7 polypeptides in the CSF and detected pro-Bmp7 at E14.5 (Fig. S1). Thus, Bmp7, when biologically active, could nurture cortical progenitor cells both via the meninges and the CSF.

To investigate whether the relative size of cortical domains is affected in the absence of Bmp7, we took advantage of a systemic *Bmp7*-deficient mouse line (Bmp7^Δ/Δ^) we previously described [21] and examined serial sections the cortex of *Bmp7*-deficient (Bmp7^Δ/Δ^) embryos. We found that the cortex of E14.5 Bmp7^Δ/Δ^ embryos was thinner with a cortical plate that appeared less clearly defined (Fig. 1C, A, A′). The differences were most prominent in the medial regions of the developing cortex, whereas more lateral regions were not significantly affected (Fig. 1C, B, B′). This microencephaly phenotype was 100% penetrant in more than 30 mutant embryos analysed and cellularity of the cortex was reduced by approximately 25% (Fig. S2). To explore whether this phenotype was due to the loss of Bmp7 specifically, we analyzed the presence of several other Bmps in the developing cortex (Fig. 1D). We found that no significant changes were observed in the protein levels of Bmp2, Bmp4, Bmp5, and Bmp6, respectively (see also legend to Fig. 1D). Thus, the absence of Bmp7 appears to have a specific effect on the developing cortex which results in the microencephaly observed.

**Figure 2:**
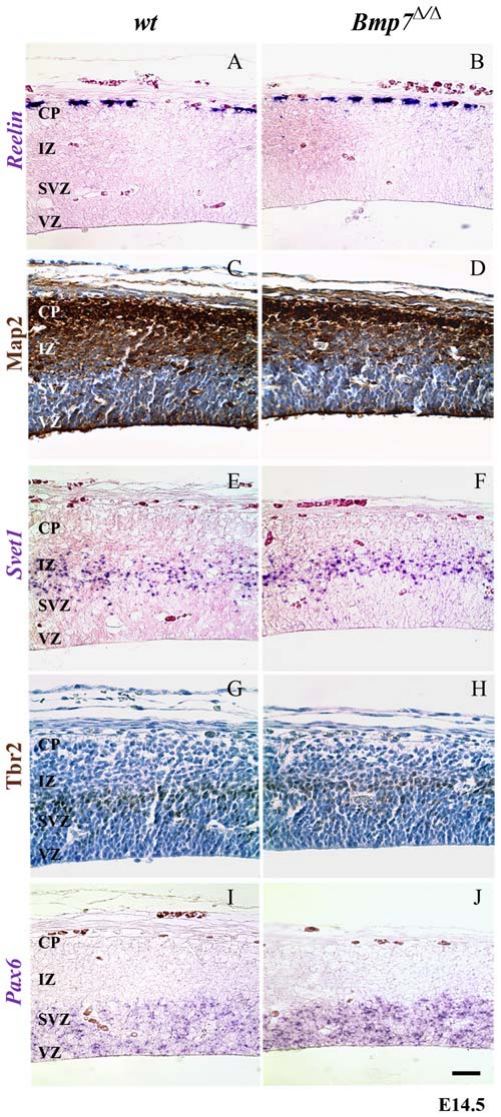
Normal layering of the E14.5 neural cortex in *Bmp7*-deficient mouse embryos. Comparison of wild-type (wt, panels A, C, E, G, I) and *Bmp7* ^Δ/Δ^ (panels B, D, F, H, J) cortical sections for expression of *Reelin* (A, B), presence of Map2 protein (C, D), Svet1 mRNA (E, F), and Tbr2 protein (G, H), and Pax6 mRNA (I, J), demonstrating their respective correct location even in the absence of *Bmp7*. The cortical plate (CP) may appear somewhat reduced in the Map2-stained sections (C, D).

The finding of microencephaly in E14.5 Bmp7^Δ/Δ^ embryos suggested that during corticogenesis Bmp7 affects cortical development in part at the level of cortical layering. We therefore examined the presence of region-specific markers known to be involved in cortical development and neuronal differentiation. Reelin mRNA, encoding a large extracellular matrix glycoprotein that marks Cajal-Retzius cells of layer I in the mouse embryo [27], [28], appeared normal in the *Bmp7* knockout embryos (Fig. 2A, B). In contrast, the expression domain of the microtubule-associated protein Map2, which marks cortical plate neurons, appeared significantly reduced (Fig. 2C, D).

A reduction in the cortical plate can be due to the reduction or complete loss of progenitor populations. We therefore also analyzed the expression of *Svet1* (Fig. 2E, F) and the presence of Tbr2 (Fig. 2G, H), which are both markers of basal progenitor cells in the subventricular zone [29], [30] and *Pax6* mRNA (Fig. 2 I, J), encoding a transcription factor that regulates the development of radial glia progenitor cells in the ventricular zone [31]. We found that all these genes/proteins were appropriately expressed/produced (Fig. 2E–J).

**Figure 3:**
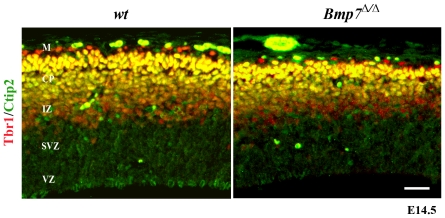
Reduced cortical plate thickness in the absence of *Bmp7*. Colocalization of Tbr1 (green) and Ctip2 (red) reveals a reduction of post-mitotic neurons in the CP marked as Tbr1/Ctip2 double positive cells (yellow). Ctip2 staining in the SVZ remained unaltered.

We then analyzed whether the reduction in cortical plate size at E14.5 specifically affected the formation of the cortical layers. Tbr1, which is present abundantly in post-mitotic neurons of the cortical plate and to a lesser extent in the marginal zone [32], was confined to a smaller area in the BMP7^Δ/Δ^ embryos indicating a reduction of the numbers of deep layer neurons (Fig. 3), in agreement with a more confined Map2 domain observed in Fig. 2D. In contrast, neither the number nor the position of the Ctip2^+^ cells present in the layer 5 of the cortical plate [33] appeared to be altered in the mutant embryos (Fig. 3). Transcripts encoding glutamic acid decarboxylase-67 (GAD67), a marker for interneurons [34], were appropriately expressed in the mutant embryos (see Fig. S3). This indicates that there was neither a defect in interneuron numbers nor their migration towards *Bmp7*-deficient cortices. These data show that lack of Bmp7 does not affect the lamination of the cortical layers *per se*, but leads to a general reduction in cortical plate thickness.

### Bmp7 regulates *Ngn2* expression in the E14.5 mouse cortex

**Figure 4:**
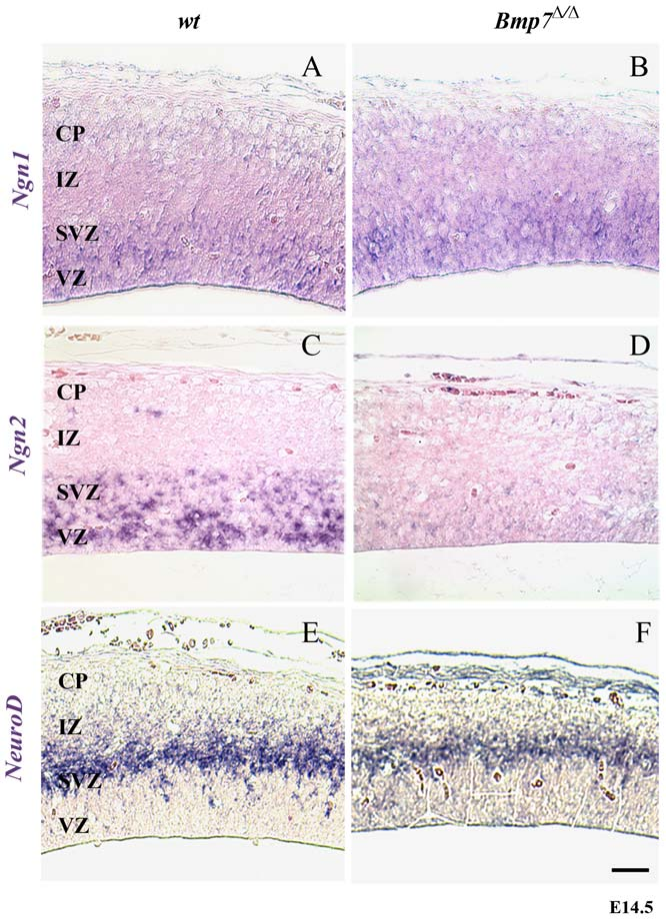
Bmp7 regulates *Ngn2* steady-state transcript levels in the E14.5 cortex. Expression of the pro-neural genes *Ngn1* (A, B), *Ngn2* (C, D), *NeuroD* (E, F) in wild-type (wt; A, C, E) and *Bmp7* ^Δ/Δ^ (B, D, F) E14.5 cortices. Whereas *Ngn1* and *NeuroD* expression appear normal, *Ngn2* is significantly reduced (transiently, see Figure S3) in the absence of *Bmp7*.

The thinner cortical plate could be due to altered differentiation of neuronal progenitors from the subventricular zone to the upper layers. As BMPs have been reported to induce the expression of the proneural gene *Ngn2* in sensory neurons [13], we investigated the expression pattern of different key proneural genes in Bmp7^Δ/Δ^ embryonic cortices. *Ngn1, Ngn2, and NeuroD* are expressed in defined dorso-ventral patterns in the subventricular (*NeuroD*) and ventricular (*Ngn1, Ngn2*) regions of the developing cortex and regulate deep layer neuron development [35], [36]. At E14.5, *Ngn1* (Fig. 4A, B) and *NeuroD* (Fig. 4E, F) expression appeared to be normal in Bmp7^Δ/Δ^ cortices, whereas *Ngn2* was almost absent (Fig. 4C, D). Interestingly, and when carefully comparing the data from various simultaneous *in situ* hybridizations on our automated platform (see Materials & Methods), which significantly contributes to the reproducibility of these experiments, *Ngn2* expression was normal at E13.5, and at E15.5 it started to reappear in the mutant embryos leading to a constantly normal expression at E17.5 (Fig. S4). This transient fluctuation of *Ngn2* expression in the absence of Bmp7 suggests that Bmp7 signalling is involved in a tight control of the steady-state levels of *Ngn2* transcripts in the developmental window between E14-15.

**Figure 5:**
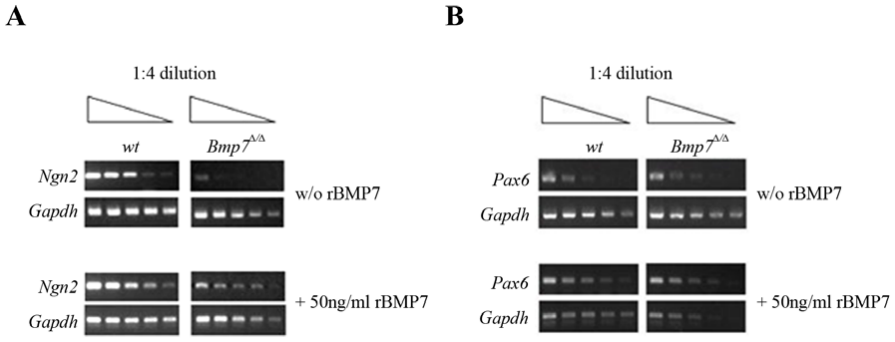
rBMP7 rescues *Ngn2* mRNA expression in cultured E14.5 *Bmp7*-deficient cortical cells. RT-PCR analysis of serial 4-fold cDNA dilutions (1, ¼, 1/16, 1/64, 1/256 cDNA input) for *Ngn2* (A) and *Pax6* (B) in isolated E14.5 wild-type (wt) or *Bmp7* ^Δ/Δ^ cortical cells cultured for 24 hrs in the absence (w/o rBMP7) or presence of BMP7 (50 ng/ml). The severely reduced *Ngn2*-expression in *Bmp7* ^Δ/Δ^ cortical cells is rescued by rBMP7 (panel A). *Ngn2* and *Pax6* expression (panel B) increase also in wild-type cells indicating that Bmp7 might be a limiting factor *in vivo*.

To establish whether Bmp7 indeed regulates *Ngn2* expression, we tested the effect of recombinant BMP7 protein (rBMP7) on isolated wild-type and Bmp7^Δ/Δ^ cortical cells *in vitro*. We found that rBMP7 increased *Ngn2* mRNA levels in wild-type cells and that it rescued *Ngn2* expression in Bmp7^Δ/Δ^ cells (Fig. 5A). Concurrently, we assayed for *Pax6* expression, a known upstream regulator of *Ngn2* [37]. The expression of *Pax6* appeared not to be altered in the BMP7^Δ/Δ^ cortex (see Fig. 2I, J and Fig. 5B). Although rBMP7 resulted in a small increase in *Pax6* expression, the increase was comparable between wild-type and *Bmp7* ^Δ/Δ^ cells (Fig. 5B). These results suggest that Bmp7 is critically needed to regulate steady-state *Ngn2* transcript levels in the E14.5 cortex, and this specifically during a narrow time window (see Fig. S4).

### BMP7 controls neural progenitor maintenance

**Figure 6:**
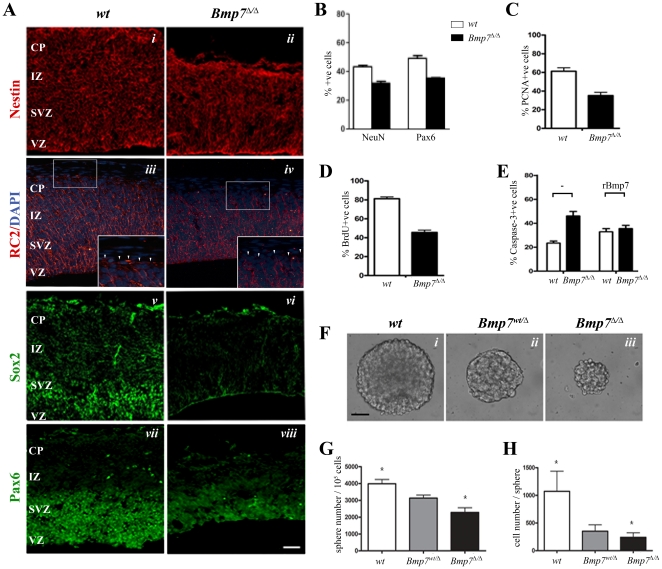
BMP7 controls radial glia survival. (A) Absence of Bmp7 affects RG and neural progenitor cells. Expression of Nestin (i, ii), RC2 (iii, iv), Sox2 (v, vi), Pax6 (vii, viii) in wt or *Bmp7* ^Δ/Δ^ E14.5 cortices. The *Bmp7* ^Δ/Δ^ cortex appears less organized (i–iv). The RGC make poor contact to the meninges (iii, iv). Sox2 and Pax6 expression are lost or diminished in the VZ/SVZ. (B) Counts of cell spreads of E14.5 cortices reveal a reduction of NeuN and Pax6-positive cells in *Bmp7* ^Δ/Δ^ cortical cells (black bar) when compared to wt littermate cells (open bar). (C, D) The number of PCNA-positive cells (C) and cells having incoporated BrdU (D) are reduced in *Bmp7* ^Δ/Δ^ cortical cells (black bar) when compared to wt littermate cells (open bar). (E) *Bmp7* ^Δ/Δ^ cortical cells (black bar) showed increased numbers of Caspase-3-positive cells when compared to wt littermate cells, which was corrected following treatment with rBMP7. (F–H) Neurospheres derived from wt, *Bmp7^wt/^* ^Δ^ heterozygote, or *Bmp7* ^Δ/Δ^ cortical cells (F) are fewer (G) and smaller (H) indicating that Bmp7 affects the survival and self-renewal properties of neural progenitor cells in the developing cortex. *p < 0.05 by Student's t test.

The thickness of the cortical plate is known to depend on the number of intermediate progenitors and neurons generated by each radial glia cell (RGC) [38], [39]. We therefore investigated whether the absence of Bmp7 also affects RGC properties specifically. The overall presence of the intermediate filament protein Nestin, which marks proliferating cells within the cortex, appeared normal, but in BMP7^Δ/Δ^ embryos displayed a disorganization of the filaments (Fig. 6A, i, ii). The disorganization, albeit less pronounced, was also observed in RGCs stained with the RC2 antibody that recognizes a RGC-specific, phosphorylated form of Nestin [40] (Fig. 6A, iii, iv). In addition, the RGCs had lost the connection with the basal membrane (Fig. 6A, iii, iv). RGC attachment to the basal membrane is important and attachment defects have been associated with reduced proliferation and survival of RGCs [41]. Hence we tested whether Sox2 and Pax6, required for the proliferation and neurogenic potential of RGCs [31], [42], were altered. Sox2 protein levels were clearly reduced (Fig. 6A, v, vi). Even though *Pax6* mRNA expression was normal in *Bmp7* ^Δ/Δ^ cortices (Fig. 2 I, J and Fig. 5B) the number of Pax6^+^ cells and possibly Pax6 protein levels appeared to be reduced (Fig. 6A, vii,viii, 6B). Similarly, the number of NeuN^+^ neurons was reduced by approximately 25%, in linear correlation with the reduction in Pax6^+^ cells (Fig. 6B). This indicated that proliferation and neurogenic potential of neural progenitor cells in BMP7^Δ/Δ^ cortices could be impaired.

To quantify the proliferation defects, we generated cell spreads from wild-type and *Bmp7* ^Δ/Δ^ E14 cortices and assayed for cell proliferation (PCNA) and cell division (BrdU). In the absence of Bmp7, we observed a 50% reduction in the number of PCNA positive (+ve) proliferating cells (Fig. 6C, D). To understand whether loss of cell proliferation was accompanied by an increase in apoptosis, we assayed the cell spreads for the presence of cleaved caspase-3. We observed a 50% increase in the number of apoptotic cells in cell spreads from *Bmp7* ^Δ/Δ^ cortices (Fig. 6E). In the same experiment, these defects were completely rescued when *Bmp7* ^Δ/Δ^ cortical cell spreads were treated with rBMP7. Taken together, the loss of Bmp7 appears to affect the survival of neural progenitor cells in the embryonic cortex.

To assess whether self-renewal of neural stem/progenitor cells was also impaired by loss of Bmp7, we analyzed the ability of wild-type and *Bmp7* ^Δ/Δ^ cortical cells to generate neurospheres *ex vivo*. In the absence of Bmp7, isolated progenitor cells from E14.5 cortex formed fewer (Fig. 6G) and smaller (Fig. 6F, iii) neurospheres suggesting that the neurosphere-forming potential and the proliferation of neural progenitor cells within these neurospheres [43], [44] were compromised. As expected the cell number per neurosphere after one week in culture was dramatically reduced in the absence of Bmp7 (Fig. 6H). Therefore, Bmp7 is not only required for the organization of RGCs but also affects the survival and self-renewal properties of neural progenitor cells in the developing cortex.

## Discussion

### Bmp7 nurtures the developing cortex via the CSF and the meninges

The development of the neocortex is an orchestrated process controlled by signals that derive from the choroid plexus, the marginal zone and the overlaying meninges. Choroid plexus derived growth factors provide a proliferative niche for neural progenitor cells [45] and surgical removal of the meninges results in severely reduced growth of the forebrain in chick [46] and mouse embryos by compromising survival and neurogenic properties of neural progenitor cells [25], [41].

Choroid plexus-derived Bmp7 is secreted into the cerebrospinal fluid (CSF) [47] and can be detected at the ventricular zone of the developing cortex. It has been shown that the CSF provides a proliferative niche for neural progenitor cells and has an age-dependent effect on cell proliferation [45]. Although this proliferative effect has been mainly attributed to IGF (Insulin Growth Factor), other signalling proteins must play a direct role as well [45]. The present data suggest that Bmp7 is one of the proteins that could act in synergy with e.g. IGF to stimulate stem cell/progenitor divisions and homeostasis in the CSF of the mouse.

Meninges are important for maintenance and homeostasis of radial glia cells (RGCs). RGCs extend their basal processes to the meningeal basement membrane and this contact is critical for the survival of these cells. Detachment of radial glia processes from the basement membrane either after meningeal ablation or genetic manipulation affecting proteins involved in the attachment process have been documented to disturb radial glia survival, morphology, and migration of newly-born neurons [41], [48]. Although these cellular events are very important for radial glia survival and function, still little is known about the meningeal signals that contribute to cortex homeostasis [25]. Our data indicate that Bmp7 represents one of these critical signals, since *Bmp7* deletion affects both the organization and function of radial glia cells *in vivo* and in cultured cortical cells. The *Bmp7* null mice exhibit in this respect striking similarities with the phenotypes observed in β1-integrin or α2 and α4-integrin deficient mouse embryos. In these mice, detachment of the radial glia processes from the meningeal basal membrane was observed [41]. Integrin levels and/or signalling might therefore be compromised in the absence of Bmp7, since – for example – Bmp7 regulates, amongst others, the expression level of the integrin-linked kinase (ILK) encoding gene [49]. ILK is a major intracellular mediator of integrin-dependent basal lamina formation [50]. In line with the critical function of these integrins, and similarly to *Bmp7*-deficient embryos, the loss of *Ilk* in the developing mouse cortex affects the organization of radial glia fibers [50]. *Ilk* deficiency also causes cobblestone lissencephaly [50], which is characterized by thicker and disorganized cortical zones. In contrast, loss of *Bmp7* results in a profound reduction of the cortical plate without concomitant loss of cortical layering. In the developing brain *Bmp7* is expressed not only in the meninges, but also in the choroid plexus.

### Bmp7 regulates cellular and neurogenic properties of neural progenitor cells

Attachment deficiencies of radial glia cells to the meninges have a direct role on proliferation and survival of neural progenitor cells. Indeed, neural progenitor cells from *Bmp7*-deficient mice exhibit higher apoptotic scores and display a deficiency in cell proliferation both *in vivo* and *ex vivo*, which could lead to the defective neurogenesis documented here. Thereby, transcriptional activators of *Ngn2* itself might be reduced in the absence of Bmp7. The best candidate is Pax6, a well-characterized regulator of *Ngn2* expression [37] and radial glia cell differentiation [31]. Though loss of Bmp7 does not appear to affect *Pax6* mRNA expression *in vivo*, Pax6 protein levels seemed reduced in *Bmp7* mutants. The number of Pax6^+^ cells also appeared reduced. Similarly, expression of *Sox2* was reduced. Sox2 is an important intrinsic regulator of cortical neurogenesis controlling the number of Pax6^+^ cells [42], [51]. These observations suggest that in the *Bmp7* mutant embryos the neural progenitor pool has impaired stem cell properties and neurogenic potential [42], [52], [53]. The presence of Tbr1-positive cells in the cortical plate of *Bmp7*-deficient mice indicates that neurogenesis *per se* was not affected. Neurosphere assays supported and confirmed this hypothesis and show that Bmp7 directly affects proliferation and survival of neural progenitor cells. However, loss of *Ngn2* expression during the narrow developmental window E14-15 in *Bmp7*-deficient cortices could be a direct consequence of the Bmp7 effects on the Pax6^+^-neural progenitor cells. The restoration of *Ngn2* expression through addition of Bmp7 to cultures of *Bmp7*-mutant cortical cells could simply be due to the rescue of progenitor cell survival, proliferation, and in consequence restoration of Pax6^+^ cell function. Thus, the reduced number of deeper layers cortical cells is a reflection of the linear relationship between the Pax6^+^-neural progenitor cells and Ngn2-dependent deep layer neurogenesis [54].

### Bmp7 exerts a temporal, specific, and non-redundant role in corticogenesis

Other genes encoding Bmps such as Bmp2, Bmp4, Bmp5, and Bmp6 are also expressed in the developing cortex. Thus, a partial functional compensation between the various Bmp family members in the developing cortex is possible. For example, loss of *Bmp4* in the forebrain has only a very mild effect on cortical formation [55]. However, our results demonstrate that Bmp7 has specific functions such as radial glia survival and Ngn2-dependent neurogenesis, which cannot be compensated by other Bmps during cortical neurogenesis. Our data also indicates that cortical neurogenesis is regulated from two independent Bmp7 sources, i.e. the meninges and the choroid plexus. It is possible that the absence of Bmp7 signalling from the meninges can affect radial glia integrity, while lack of Bmp7 from the CSF controls proliferation and Ngn2 expression. Bmp7 signals via several type I Bmp receptors, which are also high affinity receptors for other Bmp ligands. This leaves open the question of whether differential signalling through Bmp receptors by Bmp7 or the availability of a Bmp within a restricted locality determine the requirement of Bmp7 for survival, proliferation and neurogenic properties of neural progenitor cells.

## Supporting Information

Figure S1 **proBmp7 can be detected in the embryonic CSF.** Western Blot of total protein extracts of CSF aspirated from E12.5-E14.5 embryos shows the presence of proBmp7 in the CSF.
(TIF)

Figure S2 **Comparison of** ***wt*** **and** ***Bmp7*** **-deficient embryos, brain and cortices.** E14.5 (A, A′) and P0 (B, B′) embryos and E14.5 heads (C, C′) of a wt (A, B, C) or *Bmp7*-deficient (A′, B′, C′) genotype show overall comparable development. Brain development (D, D′) also appears largely normal though smaller cortices are apparent in *Bmp7*-deficient brains (D′) when compared to wt control brains (D). Cell counts of isolated cortices show an approximately 25% cell reduction. Note absent eyes in *Bmp7*-deficient embryos/mice.
(TIF)

Figure S3 **Normal interneuron development in** ***Bmp7^Δ/Δ^*** **cortices.** *GAD67*-expression was comparable between wt (A, C) and *Bmp7^Δ/Δ^* (B, D) cortices indicating normal interneuron development in the absence of *Bmp7*.
(TIF)

Figure S4 **Temporal loss of** ***Ngn2*** **in** ***Bmp7^Δ/Δ^*** **cortices at E14-15.** Expression of *Ngn2* at various developmental stages in wt (A, C, E, G) and *Bmp7^Δ/Δ^* (B, D, F, H) cortices reveals that loss of *Ngn2* expression is restricted to the developmental stages aroung E14-E15.
(TIF)
