# Supplementary figures and images for: Bmp7 Regulates the Survival, Proliferation, and Neurogenic Properties of Neural Progenitor Cells during Corticogenesis in the Mouse
Source: PLoS One. 2012 Mar 26;7(3):e34088. doi: 10.1371/journal.pone.0034088 (PMC3312908; doi:10.1371/journal.pone.0034088)

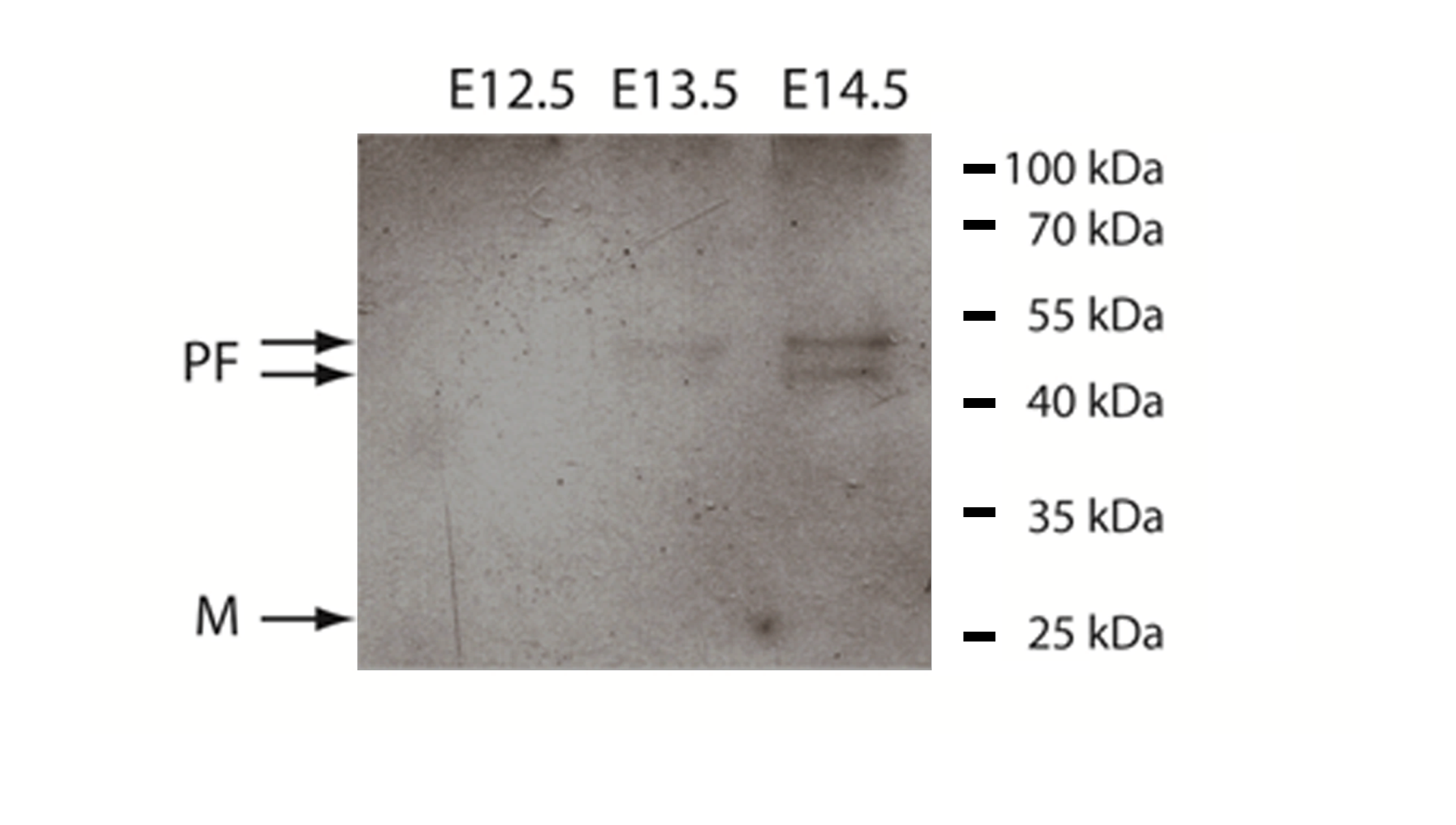

Supplement: Figure S1 — proBmp7 can be detected in the embryonic CSF. Western Blot of total protein extracts of CSF aspirated from E12.5-E14.5 embryos shows the presence of proBmp7 in the CSF. (TIF) [file pone.0034088.s001.tif]

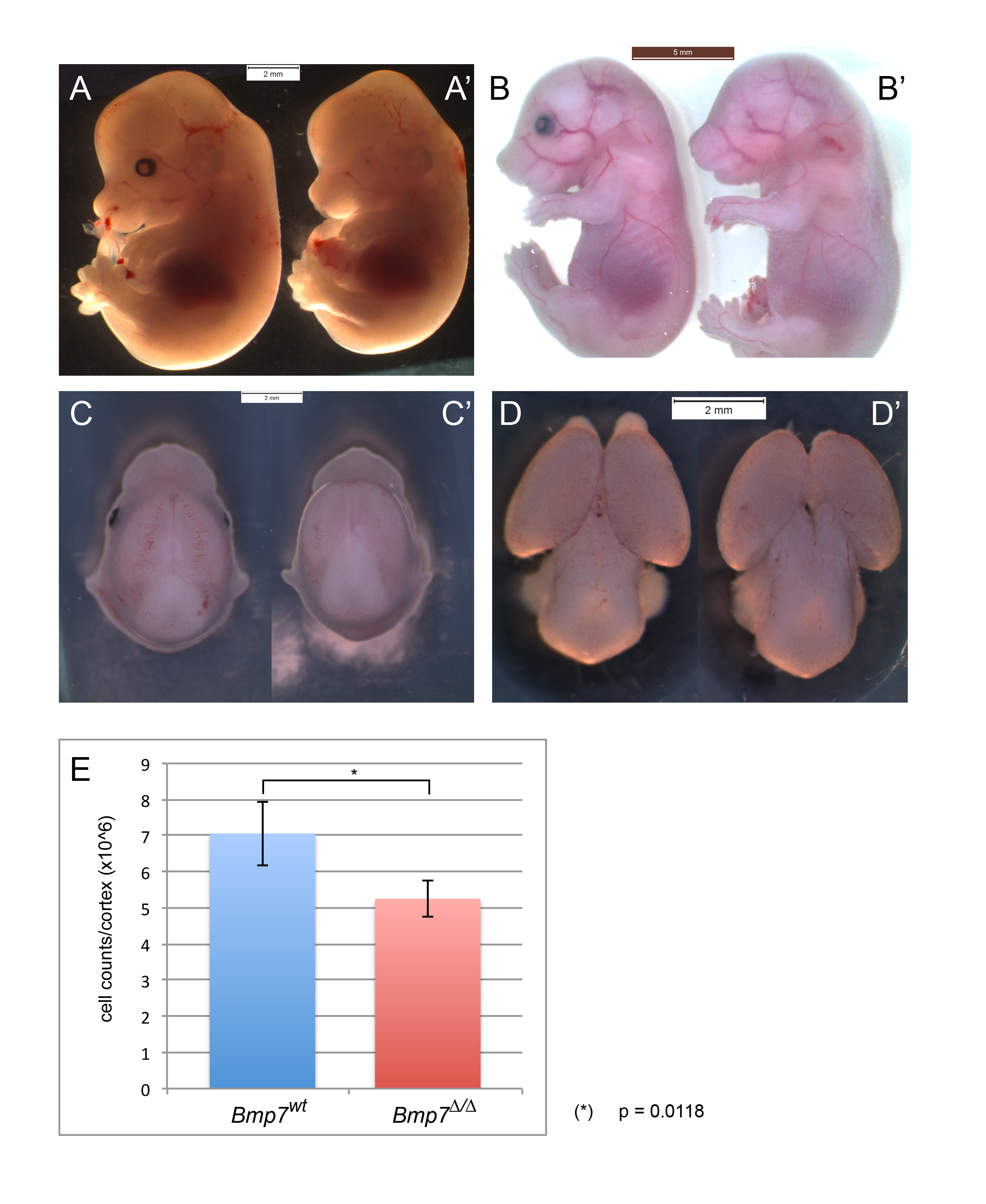

Supplement: Figure S2 — Comparison of wt and Bmp7 -deficient embryos, brain and cortices. E14.5 (A, A′) and P0 (B, B′) embryos and E14.5 heads (C, C′) of a wt (A, B, C) or Bmp7-deficient (A′, B′, C′) genotype show overall comparable development. Brain development (D, D′) also appears largely normal though smaller cortices are apparent in Bmp7-deficient brains (D′) when compared to wt control brains (D). Cell counts of isolated cortices show an approximately 25% cell reduction. Note absent eyes in Bmp7-deficient embryos/mice. (TIF) [file pone.0034088.s002.tif]

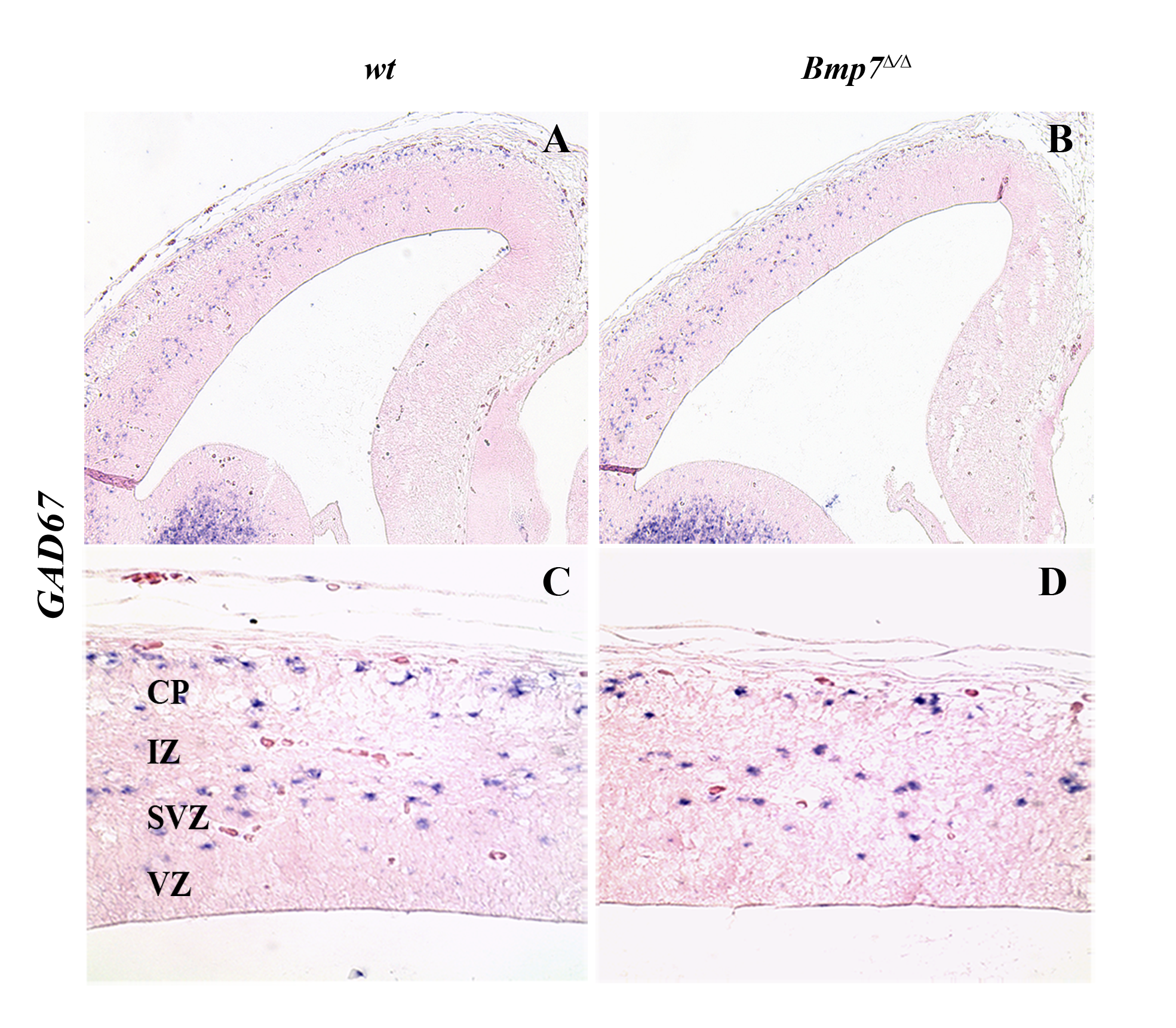

Supplement: Figure S3 — Normal interneuron development in Bmp7Δ/Δ cortices. GAD67-expression was comparable between wt (A, C) and Bmp7Δ/Δ (B, D) cortices indicating normal interneuron development in the absence of Bmp7. (TIF) [file pone.0034088.s003.tif]

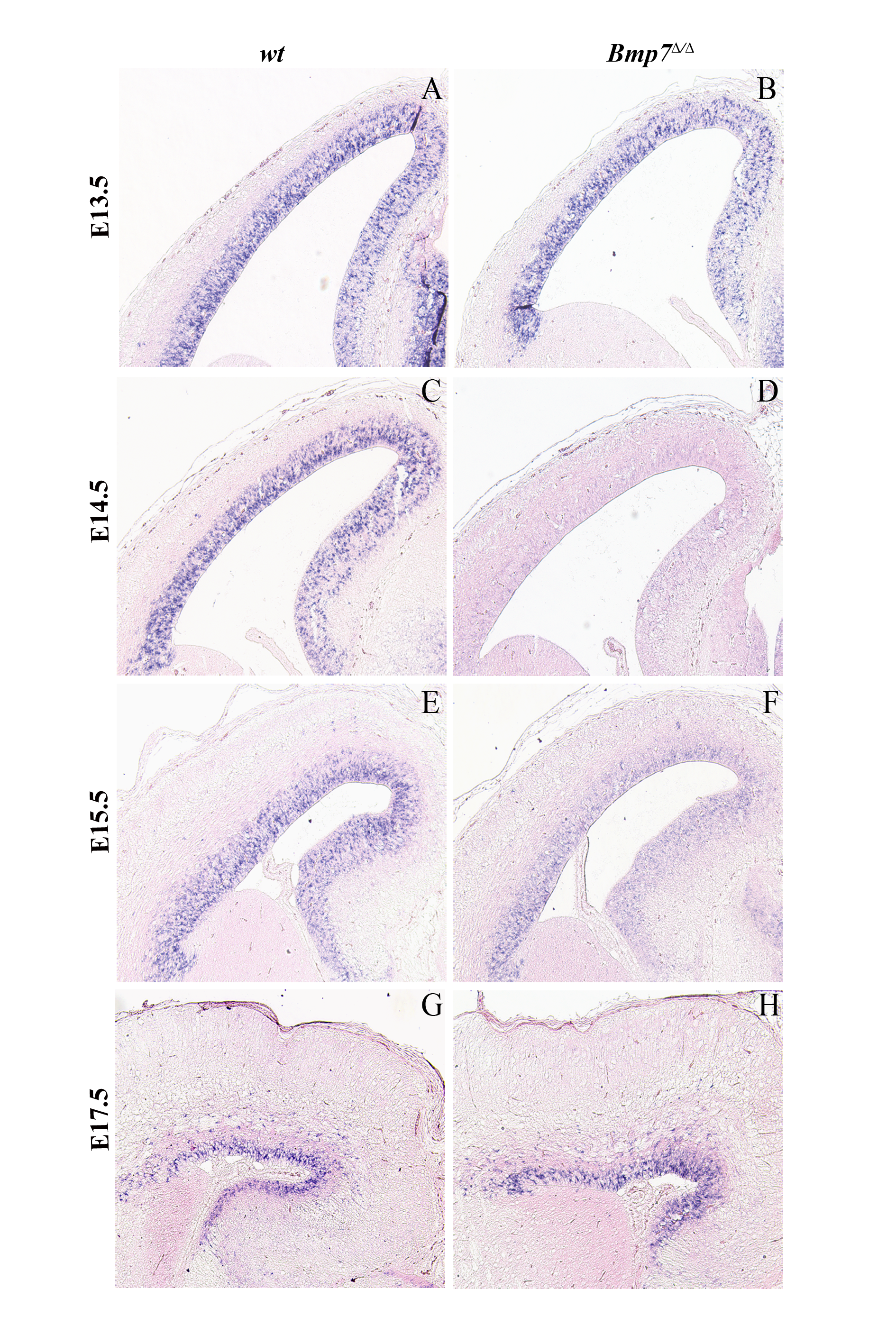

Supplement: Figure S4 — Temporal loss of Ngn2 in Bmp7Δ/Δ cortices at E14-15. Expression of Ngn2 at various developmental stages in wt (A, C, E, G) and Bmp7Δ/Δ (B, D, F, H) cortices reveals that loss of Ngn2 expression is restricted to the developmental stages aroung E14-E15. (TIF) [file pone.0034088.s004.tif]
